# Effects of Temperature and *Bacillus velezensis* on the Development, Longevity, and Reproduction of *Culex quinquefasciatus*

**DOI:** 10.3390/biology14040357

**Published:** 2025-03-30

**Authors:** Abdullah A. Alomar

**Affiliations:** Infectious Disease Vector Research Laboratory, Department of Plant Protection, College of Food and Agricultural Sciences, King Saud University, Riyadh 11451, Saudi Arabia; abdalomar@ksu.edu.sa

**Keywords:** *Bacillus velezensis*, *Culex quinquefasciatus*, biological control, temperature effects, larval development, reproductive fitness, mosquito longevity

## Abstract

Temperature is a key environmental factor that influences mosquito phenotypic traits and the effectiveness of vector control strategies. *Bacillus velezensis* (*Bv*) has shown promise as a microbial biocontrol agent due to its insecticidal properties; however, its effects on mosquitoes under different environmental conditions are still unexplored. This study investigated the effects of *Bv* (strain WHk23) exposure on the life history traits of *Culex quinquefasciatus* at two temperature conditions (20 °C and 30 °C), focusing on development, longevity, and reproductive fitness. Results showed that temperature significantly affected mosquito development and longevity, with faster development and shorter adult longevity observed at 30 °C compared to 20 °C. Exposure to *Bv* further accelerated larval development and reduced adult emergence, with the effects being more pronounced at 30 °C than at 20 °C. Exposure to *Bv* reduced adult longevity regardless of temperature. In addition, *Bv*-exposed females had larger body sizes but lower fecundity and fertility, suggesting that *Bv* exposure may cause physiological stress that disrupts reproductive processes. These findings highlight the importance of considering environmental factors in mosquito control programs while reinforcing the efficacy of *Bv* as a sustainable biocontrol agent under a variety of environmental conditions.

## Simple Summary

Human diseases transmitted by mosquito vectors are a major public health concern. Controlling mosquito populations is challenging, especially when some species become resistant to common chemical insecticides. This study explored the effects of the naturally occurring bacterium *Bacillus velezensis* (*Bv*) under two different temperatures (20 °C and 30 °C), on the development, longevity, and reproduction of *Culex quinquefasciatus*. The results showed that mosquitoes developed faster and had shorter lifespans at higher temperatures. Exposure to the bacterium further accelerated mosquito development and reduced the number of adult mosquitoes, with the effect being more pronounced at higher temperatures. Despite being larger, *Bv*-exposed mosquitoes laid fewer eggs and had lower reproductive success. This study highlights the importance of considering environmental variations when evaluating potential biocontrol agents for mosquito control and support efforts to develop safer alternatives to chemical insecticides.

## 1. Introduction

Mosquitoes are among the most important vectors of human diseases, such as chikungunya virus, dengue virus, and Zika virus. For decades, chemical insecticides, such as pyrethroids, organophosphates, and carbamates have been the primary tools for mosquito control, effectively reducing mosquito populations and limiting disease transmission [1,2]. However, the extensive and prolonged use of these chemicals has led to several challenges, including the emergence of insecticide resistance in mosquito populations, environmental contamination, and adverse effects on non-target organisms [1,3]. Thus, there is an urgent need for sustainable and environmentally friendly mosquito control strategies that minimize environmental impacts while effectively managing vector populations.

Biological control strategies offer sustainable alternatives to chemical insecticides, effectively addressing the problems of resistance and environmental impact. The most effective agents include *Bacillus thuringiensis* subsp. *israelensis* (*Bti*) and *Lysinibacillus sphaericus*, which selectively target mosquito larvae while minimizing impacts on non-target organisms [4]. However, their effectiveness is significantly affected by environmental factors, particularly temperature. Studies indicate that temperature influences the toxicity of *Bti* and other microbial insecticides by affecting bacterial sporogenization, toxin stability, and mosquito susceptibility, potentially limiting their effectiveness under different climatic conditions [4,5].

An emerging and promising microbial agent, *Bacillus velezensis* (*Bv*), has shown potential in mosquito control due to its ability to produce bioactive compounds with insecticidal properties [6,7,8,9]. Despite its promising characteristics, little is known about how environmental factors affect the efficacy of *Bv* against mosquito populations. Mosquito life history traits, including larval development, adult emergence, longevity, and reproductive fitness, are critical determinants of mosquito population dynamics and disease transmission risk. Because these traits are highly temperature-dependent, understanding their interaction with *Bv* exposure across different temperatures is essential to optimize its use under variable ecological conditions [10].

Evaluating these developmental and reproductive characteristics can reveal the sublethal impacts of microbial biocontrol agents, which might not cause immediate mortality but can significantly reduce mosquito fitness and population viability over time. Addressing this knowledge gap is important to accurately predict the efficacy of *Bv* under different environmental conditions, especially in regions experiencing temperature variations due to climate change. Therefore, this study aims to determine the effects of exposure to *Bv* on mosquito life history traits under two different environmental temperature conditions (20 °C and 30 °C). Specifically, assessing larval development, adult emergence, female longevity, and reproductive success following bacterial exposure. The results will contribute to a better understanding of how temperature influences *Bv* efficacy and will inform integrated vector management strategies that consider climate variability to enhance mosquito control effectiveness.

## 2. Materials and Methods

### 2.1. Mosquito Rearing

A laboratory colony of *Cx. quinquefasciatus* was maintained under controlled conditions of 26 ± 1 °C, 60 ± 10% relative humidity, and a 12 h light–dark cycle. Egg rafts were collected from adult cages using water-filled cups and transferred to larval rearing trays. Larvae were fed Tetramin fish food (Tetrawerke, Melle, Germany) until pupation. Newly formed pupae were placed in water-filled cups and transferred to insect-rearing cages (30 cm^3^, BioQuip Products, Compton, CA, USA) until adult emergence. Adult mosquitoes were fed 10% sucrose solution via dental cotton balls, and females were offered defibrinated chicken blood weekly to lay eggs using a Hemotek membrane feeding system (Hemotek, Blackburn, UK).

### 2.2. Crude Toxin Preparation and Toxicity

*Bacillus velezensis* strain WHk23 was isolated from soil samples collected in Wadi Hanifah, Riyadh, as previously described [7]. Following the previous methodology, bacteria were cultured in 300 mL of NB2 medium in a sterile 1 L flask and incubated at 30 °C for 48 h with constant shaking (200 rpm). The bacterial culture was centrifuged at 13,000× *g* for 10 min at 4 °C, and the supernatant was collected. To precipitate the crude toxin, the supernatant was acidified to a pH of 2 with 6 N HCl and stored at 4 °C overnight. The precipitate was collected by centrifugation at 9000× *g* for 30 min at 4 °C, resuspended in dechlorinated water, and neutralized to a pH of 7 with 1 N NaOH. The resulting crude toxin was lyophilized, weighed, and stored at 4 °C until subsequent bioassays. A stock solution of crude toxin (10 mg/mL) was prepared in dechlorinated water and serially diluted for a bioassay test of mosquito larvae. Third-instar *Cx. quinquefasciatus* larvae were exposed to 10 different concentrations (from 0 to 100 µg/mL), and each bioassay was performed in plastic cups containing 200 mL of dechlorinated water and twenty larvae per cup, with three replicates per concentration. The bioassay was performed at 26 °C, and larval mortality was recorded after 24 h of exposure. Based on the preliminary test results of probit analysis, an LC_30_ concentration (17.92 µg/mL) was purposefully selected, assuming that exposure to a low concentration would cause both lethal and sublethal effects on mosquito life history traits (e.g., growth and fitness).

### 2.3. Experimental Treatments

**Figure 1:**
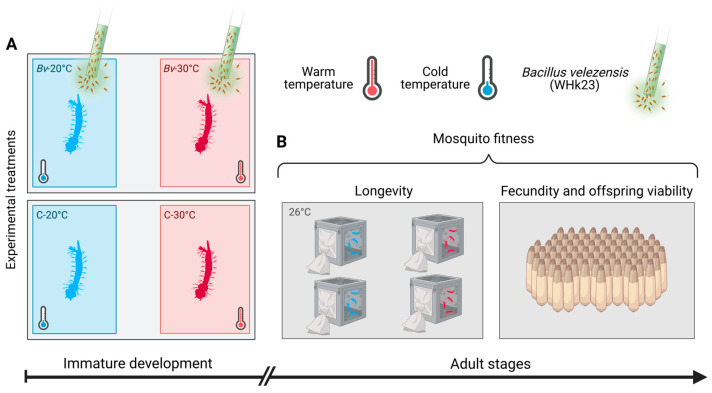
Experimental workflow. Larvae of *Cx. quinquefasciatus* were subject to four levels of treatments (Control-20 °C, *Bv*-20 °C, Control-30 °C, and *Bv*-30 °C) (**A**). A low concentration of *Bv* crude toxin that approximately causes 30% mortality among larvae was applied to the *Bv*-20 °C and *Bv*-30 °C treatments after larvae reached third instars, whereas the control groups received no treatment. Following the exposure at immature stages, emerging adult fitness (e.g., longevity and fecundity) was determined for mosquitoes from each experimental treatment (**B**).

Three hundred mosquito larvae (<24 h old) were counted and placed in experimental trays containing 1.5 L of dechlorinated water with 0.2 g of larval food. The experimental trays were divided into two treatment groups, which were incubated at 20 °C and 30 °C. The experimental trays, serving as replicates, were assigned to one of four treatment groups: Control-20 °C, *Bv*-20 °C, Control-30 °C, and *Bv*-30 °C, with each treatment replicated three times. The low concentration of *Bv* that approximately causes 30% larval mortality was applied to larvae in the *Bv*-20 °C and *Bv*-30 °C treatments after they reached third instars, whereas the control groups received no *Bv* treatment (Figure 1).

Trays were monitored daily, and newly emerged pupae were transferred to water-filled vials for adult emergence. After emergence, adults were transferred to cages with mesh and given continuous access to cotton balls soaked in 10% sucrose and maintained at 26 °C until death. Development time (from egg hatch to emergence, measured in days), total adult emergence rate in percentage, and adult female longevity (from emergence to death, measured in days) (*N* = 50 per replicate) were determined for each treatment group.

### 2.4. Fecundity, Fertility, and Wing Size

To assess the effects of treatments on mosquito fecundity, adult males and females were housed in cages for 5–8 days to allow for mating. Mosquitoes were deprived of 10% sucrose overnight and then offered to feed on defibrinated chicken blood using a Hemotek membrane blood feeder (Hemotek, Blackburn, UK) pre-heated to 37 °C. Mosquitoes were then cold anesthetized, and only fully engorged females (*N* = 30) from each treatment group were individually placed in new cup cages with mesh screens and given 10% sucrose via cotton balls placed on top of the cages. Each cage contained an oviposition cup filled with water. The total number of eggs laid by a female was recorded as a measure of fecundity. Hatching success (fertility) was determined by counting the number of neonates that hatched from each egg raft. The body size of female mosquitoes was estimated using wing length as a proxy. Wings were dissected from a group of females (*N* = 30) from each treatment group, placed on glass slides with double-sided tape, and their length in millimeters was measured from the alular notch to the distal margin, excluding the fringe, using a calibrated dissecting microscope equipped with an ocular micrometer.

### 2.5. Statistical Analysis

Data for the effects of treatments on life history traits, including development time, emergence, reproduction, and wing length, were analyzed using separate two-way analyses of variance (ANOVA) followed by Tukey’s post hoc tests for multiple comparisons. Adult female survival was analyzed using a Cox proportional hazards regression analysis of survival data. Statistical analyses were performed using GraphPad Prism 10 (GraphPad Software, San Diego, CA, USA). *p* values of <0.05 were considered statistically significant.

## 3. Results

**Figure 2:**
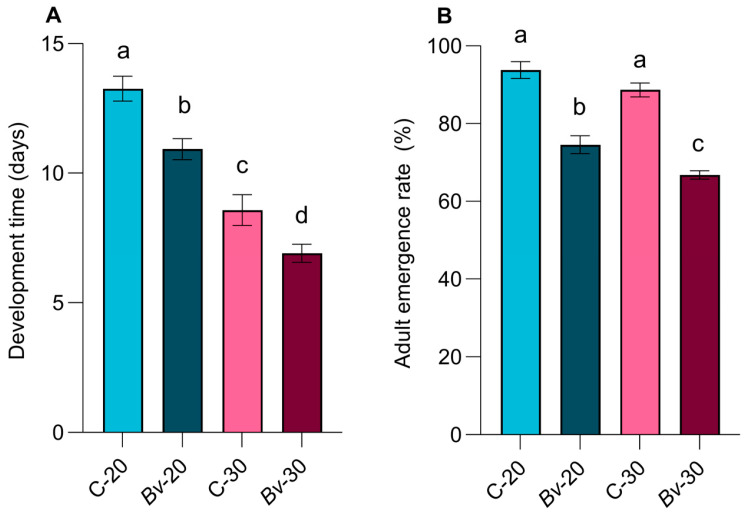
Treatment effects on immature development time (**A**) and adult emergence rate (**B**). Bars represent means ± standard error of the means. Different letters indicate statistically significant differences between treatment groups (*p* < 0.05).

Temperature significantly affected mosquito development time, with mosquitoes reared at 30 °C developing faster than those reared at 20 °C (F = 70.73, *p* < 0.0001). Furthermore, exposure to *Bv* significantly further accelerated development at both temperatures, leading to earlier adult emergence compared with control groups (F = 18.74, *p* = 0.0002) (Figure 2).

**Figure 3:**
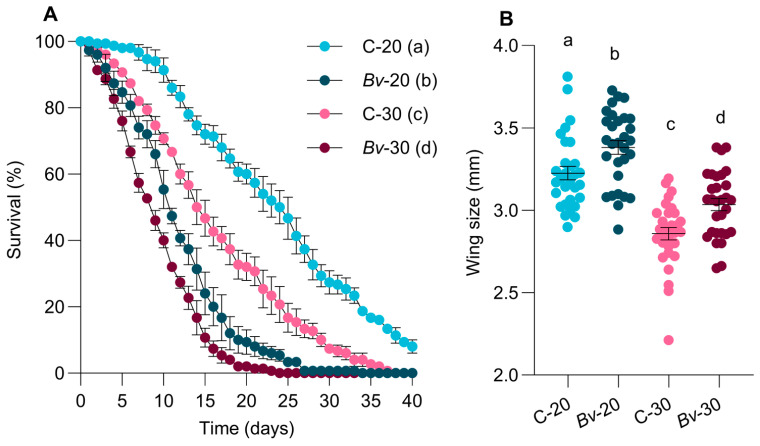
Survival curves of female mosquitoes following the exposure at immature stages. Data shown represent the means ± standard error of the means (**A**). Female wing length (**B**). Horizontal lines indicate means ± standard error of the means. In panel (**B**), each dot represents data from an individual mosquito. Different letters next to treatment names indicate statistically significant differences between treatment groups (*p* < 0.05).

The adult emergence rate was significantly reduced in larvae exposed to *Bv* (F = 1014, *p* < 0.0001), with the inhibitory effect being more pronounced at 30 °C than at 20 °C (F = 222.5, *p* = 0.0009) (Figure 2). Mosquito development and emergence were significantly influenced by temperature and *Bv* exposure, but their interaction was not statistically significant (*p* = 0.07). The regression analysis of survival data showed that treatments had significant effects on female longevity (χ^2^ = 229.3, *p* < 0.0001). Adult longevity was shorter in mosquitoes exposed to *Bv* than in control groups, and mosquitoes reared at 30 °C showed lower survival than those reared at 20 °C (Figure 3A).

**Figure 4:**
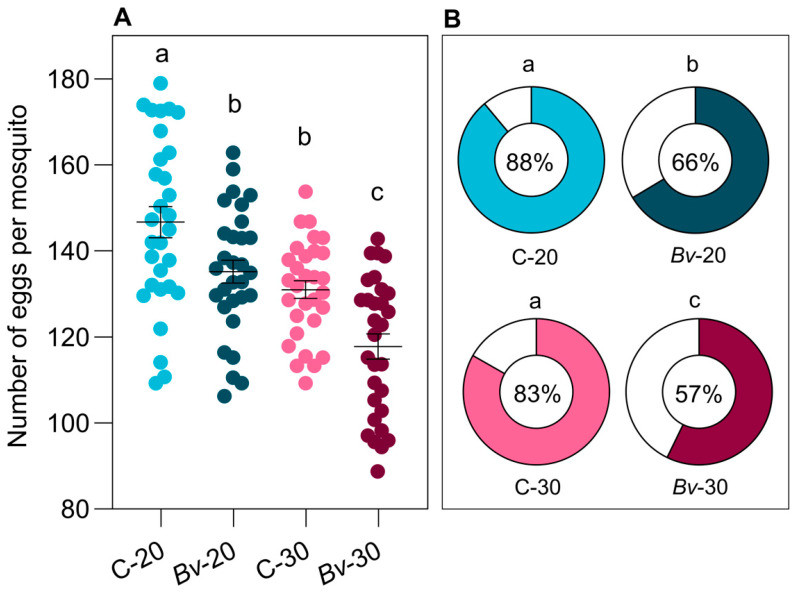
Fecundity (**A**) and fertility (**B**) of mosquitoes for each treatment group. Horizontal lines indicate means ± standard error of the means. In panel (**A**), each dot represents a single mosquito sample. In panel (**B**), the filled areas on the donut charts and the central numbers represent the percentage of hatchability (fertility). Different letters indicate statistically significant differences between treatment groups (*p* < 0.05).

Female mosquitoes exposed to *Bv* were larger in body size than those in the control groups, regardless of temperature (Figure 3B). However, *Bv*-exposed females had lower fecundity than their control counterparts (Figure 4A).

Temperature also affected fecundity, with females reared at 20 °C producing more eggs and showing greater fertility than those reared at 30 °C (F = 8232, *p* < 0.0001). Nevertheless, *Bv* exposure consistently reduced fecundity and fertility at both temperatures, regardless of female body size (Figure 4A,B). Higher temperatures increased the effects of *Bv*, leading to greater reductions in emergence, longevity, and reproductive success at 30 °C compared to 20 °C. Mosquito longevity, body size, and reproductive success were significantly influenced by temperature and *Bv* exposure, but not their interaction (*p* > 0.05).

## 4. Discussion

Temperature plays a key role in modulating insecticide toxicity against mosquitoes, influencing physiological responses and mortality rates [10]. In this study, the exposure to *Bv* further reduced adult development and emergence at 30 °C compared to 20 °C, indicating a temperature-dependent increase in larvicidal efficacy. This finding is consistent with previous studies of *Bti* and other insecticides which found that higher temperatures increase the toxicity of insecticides, suggested to be due to accelerating metabolic processes, increasing toxin uptake, and decreasing detoxification efficiency [4,11]. Similarly, Walker [12] reported that *Bti* toxicity increased with temperature, as mosquito larvae showed higher mortality rates under warmer conditions, while Nayar et al. [13] found that *Ae. taeniorhynchus* and *Cx. nigripalpus* larvae were more susceptible to *Bti* at 35 °C compared to 15 °C, supporting the positive correlation effect between temperature and insecticide efficacy. Consistent with these findings, larval exposure to malathion at higher temperatures exacerbated mortality rates in *Cx. restuans* and *Ae. albopictus* [14]. Pyriproxyfen inhibited pupal–adult transformation of *Ae. aegypti* more effectively at 30 °C than at 20 °C, underlining that elevated temperatures increase the toxicity of insect growth regulators [15]. These observations further support the temperature-dependent increase in insecticide efficacy for various biocontrol agents. However, not all insecticides follow this trend. For instance, Whiten and Peterson [16] reported that permethrin-induced mortality in *Ae. aegypti* decreased with increasing temperature from 16 °C to 30 °C, suggesting reduced efficacy at higher temperatures. These contrasting results demonstrate that temperature–insecticide interactions are complex, highlighting the need to consider temperature variations when planning vector control programs. Understanding how temperature affects insecticide efficacy is critical for effective mosquito control, especially under changing climatic conditions.

This study showed that temperature significantly affected the mosquito development time, with the faster development and production of smaller adults observed at 30 °C compared to 20 °C, which is consistent with previous findings on mosquito growth rates under different larval temperature conditions [17,18,19]. Exposure to *Bv* further accelerated development at both temperatures, leading to earlier adult emergence compared to control groups, in agreement with previous studies [20,21]. Although this study did not determine the exact mechanism behind this observation, one possibility is that mortality among immature stages due to *Bv* exposure reduced competition, allowing surviving larvae to grow faster and emerge as adults more quickly. This may explain the observed faster development despite the trend toward smaller adult size at higher temperatures. Similar mechanisms have been suggested in other studies in which exposure to insecticides during larval stages resulted in shorter development times and altered adult body sizes in mosquitoes [20,22]. These results suggest that *Bv* affects not only mosquito survival but also key life history traits, potentially influencing population dynamics and vector fitness.

Despite their larger body size, females exposed to *Bv* did not show a fitness advantage in terms of reproductive success, as their fecundity was significantly lower than that of control females. This finding contradicts the common assumption that larger mosquitoes produce more eggs, suggesting that *Bv* exposure imposes physiological costs that negatively impact reproductive fitness [23,24]. Similar trends have been observed in other studies, where the exposure of larvae to insecticides led to increased adult body size but decreased fecundity and fertility. For example, Gowelo et al. [25] reported that *An. coluzzii* exposed to *Bti* as larvae developed larger wings but showed a decreasing trend in fecundity with increasing concentrations. Similarly, Bahrami et al. [26] found that *Ae. albopictus* exposed to *Bti* during larval development experienced significant reductions in egg production. These results suggest that sublethal exposure to microbial larvicides during development may impose physiological trade-offs that extend into adulthood, potentially reducing reproductive success. Temperature also affected reproductive output, with females reared at 20 °C producing more eggs and exhibiting higher fecundity compared to those reared at 30 °C, which is consistent with previous studies. However, *Bv* exposure consistently reduced fecundity and fertility at both temperatures, regardless of female body size. This indicates that *Bv* treatment causes physiological stress that reduces the reproductive benefits typically associated with larger body size. Further research is needed to elucidate the mechanisms underlying the interaction between temperature and *Bv* exposure in shaping mosquito life history traits and fecundity. Understanding these effects is essential to assessing the broader implications of *Bv* as a biological agent and its role in integrated mosquito management strategies, particularly under variable environmental conditions.

Adult mosquito longevity was dependent on both larval temperature and *Bv* exposure, with mosquitoes reared at higher temperatures showing reduced survival compared to those reared at lower temperatures. Specifically, mosquitoes that developed at 30 °C had shorter adult lifespans than those reared at 20 °C, indicating a negative correlation effect between larval temperature and adult longevity. This trend is consistent with previous studies, showing that warmer larval environments accelerate development but reduce adult survival [19,27]. Furthermore, mosquitoes exposed to *Bv* during the larval stage showed lower adult survival at both temperatures compared to their unexposed counterparts, suggesting that microbial exposure imposes sublethal costs that persist into adulthood [25,26]. Because female longevity is a key determinant of vectorial capacity, shortened longevity due to higher temperatures and *Bv* exposure during larval stages is expected to reduce vectorial capacity and disease transmission risk.

## 5. Conclusions

This study highlights the role of temperature and microbial larvicide exposure in shaping mosquito life history traits and vectorial capacity parameters. Higher temperatures accelerated development but reduced adult longevity and reproductive success, while *Bv* exposure further exacerbated these effects, leading to earlier emergence but lower fecundity, fertility, and longevity. These results suggest that *Bv* may enhance control efforts by reducing mosquito longevity and reproductive potential, ultimately reducing disease transmission. These results underscore the importance of considering temperature variations when designing mosquito control strategies and predicting disease transmission under climate change scenarios.

## Data Availability

The data presented in this study are available from the corresponding author on a reasonable request.
